# *Klebsiella pneumoniae* Antimicrobial Drug Resistance, United States, 1998–2010

**DOI:** 10.3201/eid1901.120310

**Published:** 2013-01

**Authors:** Guillermo V. Sanchez, Ronald N. Master, Richard B. Clark, Madiha Fyyaz, Padmaraj Duvvuri, Gupta Ekta, Jose Bordon

**Affiliations:** Author affiliations: George Washington University, Washington, DC, USA (G.V. Sanchez);; Quest Diagnostics Nichols Institute, Chantilly, Virginia, USA (R.N. Master, R.B. Clark);; Providence Hospital, Washington (M. Fyyaz, P. Duvvuri, G. Ekta, J. Bordon)

**Keywords:** Klebsiella pneumoniae, CRKP, cross-resistant Klebsiella pneumoniae, antimicrobial resistance, cross resistance, Surveillance, United States, bacteria

## Abstract

We studied antimicrobial-resistant *Klebsiella pneumoniae* for 1998–2010 by using data from The Surveillance Network. Susceptibility results (n = 3,132,354) demonstrated significant increases in resistance to all antimicrobial drugs studied, except tetracycline. Cross-resistance among carbapenem-resistant *K. pneumoniae* was lower for tetracycline and amikacin.

*Klebsiella* spp. are among the most common pathogens isolated in intensive care units (ICUs), and *K. pneumoniae* is the most frequently encountered carbapenemase-producing *Enterobacteriaceae* (*1*). Increasing antimicrobial drug resistance, including carbapenem-resistant *K. pneumoniae* (CRKP), accounts for substantial increases in illness and death (*1*). Few antimicrobial therapy options exist for infections caused by CRKP (*2*).

The emergence of *K. pneumoniae* resistance to carbapenems is well documented (*3*). However, few studies have analyzed the trends and prevalence of in vitro *K. pneumoniae* antimicrobial drug resistance since carbapenem resistance emerged in the United States during the late 1990s (*4*). Furthermore, few investigations have examined antimicrobial drug resistance with regard to specimen source or cross-resistance patterns among CRKP.

We examined the prevalence of *K. pneumoniae* antimicrobial drug resistance in US inpatients using a large national surveillance system. Our objectives were to analyze *K. pneumoniae* antimicrobial drug resistance among US inpatients, resistance patterns by specimen source, and cross-resistance among imipenem-resistant *K. pneumoniae* isolates.

## The Study

We examined inpatients’ antimicrobial susceptibility test results from The Surveillance Network (TSN) Database-USA (Eurofins Medinet, Chantilly, VA, USA) for 1998–2010. TSN is a nationally representative repository of antimicrobial susceptibility results from ≈200 community, government, and university health care institutions in the United States and has been used in investigations of trends and prevalences of antimicrobial drug resistance (*5*). Susceptibility testing of isolates is conducted onsite by using Food and Drug Administration (FDA)–approved testing methods and interpreted by using Clinical Laboratory Standards Institute breakpoint criteria for all agents except tigecycline, for which FDA breakpoints were used. Details of quality control in TSN Database-USA have been described (*6*). No institutional review board approval was needed for this research because no personal identifying information was collected.

*K. pneumoniae* antimicrobial susceptibility results were stratified by specimen source (blood, sputum, urine, and wounds). Imipenem-resistant *K. pneumoniae* isolates from 2010 were examined for cross-resistance to other antimicrobial agents and prevalence in ICU versus non-ICU settings. We used χ^2^ testing to determine whether changes in *K. pneumoniae* antimicrobial drug resistance were statistically significant from 2000 to 2010 and whether 2010 antimicrobial drug resistance differed by specimen source. The α level was set at 0.05. Analyses were performed by using R version 2.11.0 (www.r-project.org).

**Figure 1:**
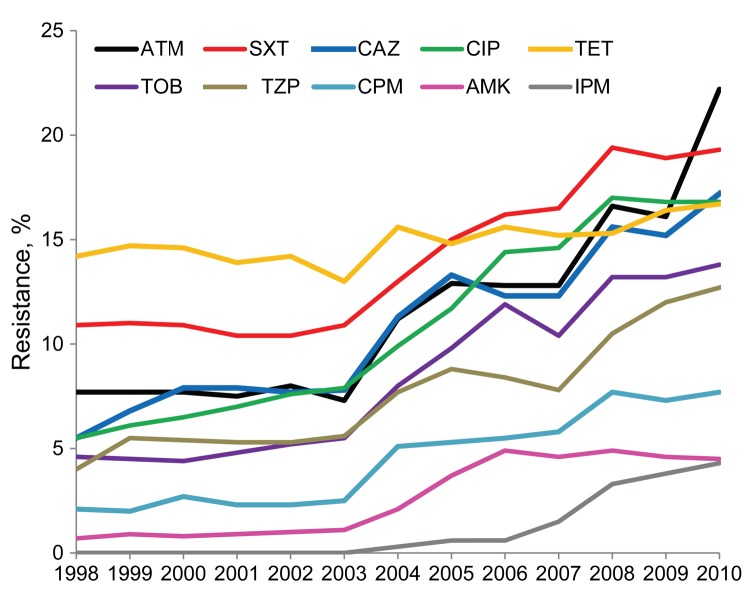
*Klebsiella pneumoniae* antimicrobial drug resistance, United States, 1998–2010. ATM, aztreonam; SXT, trimethoprim/sulfamethoxazole; CAZ, ceftazidime; CIP, ciprofloxacin; TET, tetracycline; TOB, tobramycin; TZP, piperacillin/tazobactam; CPM, cefepime; AMK, amikacin; IPM, imipenem. Ceftriaxone and gentamicin were not included for better data presentation.

We analyzed a total of 3,132,354 *K. pneumoniae* antimicrobial susceptibility results for 1998–2010 (Table 1). Statistically significant increases in antimicrobial drug resistance to all agents (p<0.0001) except tetracycline (p = 0.0745) (Figure 1) were observed. Resistance to imipenem first appeared in TSN Database-USA in 2004 and rose gradually to 4.3% by the end of our study period. In 2010, *K. pneumoniae* resistance to tigecycline was 2.6% (data not shown). The largest increases in antimicrobial drug resistance from 1998 to 2010 were observed for aztreonam (7.7% to 22.2%), ceftazidime (5.5% to 17.2%), and ciprofloxacin (5.5% to 16.8%). Changes in resistance were smaller for tetracycline (14.2% to 16.7%) and amikacin (0.7% to 4.5%).

**Table 1:** *Klebsiella pneumoniae* antimicrobial drug resistance among inpatients, by year, United States, 1998–2010*

Antimicrobial drug	No.	1998	1999	2000	2001	2002	2003	2004	2005	2006	2007	2008	2009	2010	Total change†
TET	80,862	14.2	14.7	14.6	13.9	14.2	13	15.6	14.8	15.6	15.2	15.3	16.4	16.7	2.5
AMK	232,933	0.7	0.9	0.8	0.9	1	1.1	2.1	3.7	4.9	4.6	4.9	4.6	4.5	3.8
IPM	259,589	0	0	0	0	0	0	0.3	0.6	0.6	1.5	3.3	3.8	4.3	4.3
GEN	344,597	4.9	4.6	4.6	5.2	5.5	5.6	6.9	8.3	8.4	7.8	8.7	9.1	9.2	4.3
CPM	211,134	2.1	2	2.7	2.3	2.3	2.5	5.1	5.3	5.5	5.8	7.7	7.3	7.7	5.6
TMP/SXT	344,522	10.9	11	10.9	10.4	10.4	10.9	13	15	16.2	16.5	19.4	18.9	19.3	8.4
TZP	250,554	4	5.5	5.4	5.3	5.3	5.6	7.7	8.8	8.4	7.8	10.5	12	12.7	8.7
TOB	289,287	4.6	4.5	4.4	4.8	5.2	5.5	8	9.8	11.9	10.4	13.2	13.2	13.8	9.2
CRO	287,091	1.8	2.7	3.2	2.9	2.6	3.1	5.7	6.9	8.1	8.7	11.1	11.2	12.1	10.3
CIP	306,348	5.5	6.1	6.5	7	7.6	7.9	9.9	11.7	14.4	14.6	17	16.8	16.8	11.3
CAZ	248,004	5.5	6.8	7.9	7.9	7.7	7.8	11.3	13.3	12.3	12.3	15.6	15.2	17.2	11.7
ATM	184,981	7.7	7.7	7.7	7.5	8	7.3	11.2	12.9	12.8	12.8	16.6	16.1	22.2	14.5

*TET, tetracycline; AMK, amikacin; IPM, imipenem; GEN, gentamicin; CPM, cefepime; TMP/SXT, trimethoprim/sulfamethoxazole; TZP, piperacillin/tazobactam; TOB, tobramycin; CRO, ceftriaxone; CIP, ciprofloxacin; CAZ, ceftazidime; ATM, aztreonam. †Change from 1998 to 2010. p<0.0001 for all antimicrobial agents except tetracycline (p = 0.0745).

In 2010, isolates from the lower respiratory tract showed higher levels of resistance than did isolates from urine for all antimicrobial agents (p<0.0001) except tetracycline (p = 0.54) (Table 2). CRKP was more prevalent in ICU settings than in non-ICU settings (6.3% vs. 3.8%, respectively) (Technical Appendix).

**Table 2:** *Klebsiella pneumoniae* antimicrobial drug resistance rates, United States, 2010*

Source, no. samples	AMK	GEN	TOB	TZP	ATM	IPM	CAZ	CRO	CPM	TMP/SXT	CIP	TET
All, 187,359	4.5	9.2	13.8	12.7	22.2	4.3	17.2	12.1	7.7	19.3	16.8	16.7
Blood, 20,185	4.2	10.6	14.9	15	23.6	5.9	19.2	13.4	7.1	21.3	18.9	17.1
Urine, 112,567	4.1	7.8	12.4	10.6	21	3.8	15.1	10.9	7.1	18.3	15.1	17.3
Wound, 22,225	4.5	10.1	14.4	14.7	20.9	5	17.5	12.2	8.3	19.5	16.8	16.4
Respiratory, 32,382	5.8	11.1	15.8	16.4	24.5	4.7	21.3	15.5	9.9	20.2	20	15.5

*AMK, amikacin; GEN, gentamicin; TOB, tobramycin; TZP, piperacillin/tazobactam; ATM, aztreonam; IPM, imipenem; CAZ, ceftazidime; CRO, ceftriaxone; CPM, cefepime; TMP/SXT, trimethoprim/sulfamethoxazole; CIP, ciprofloxacin; TET, tetracycline.

**Figure 2:**
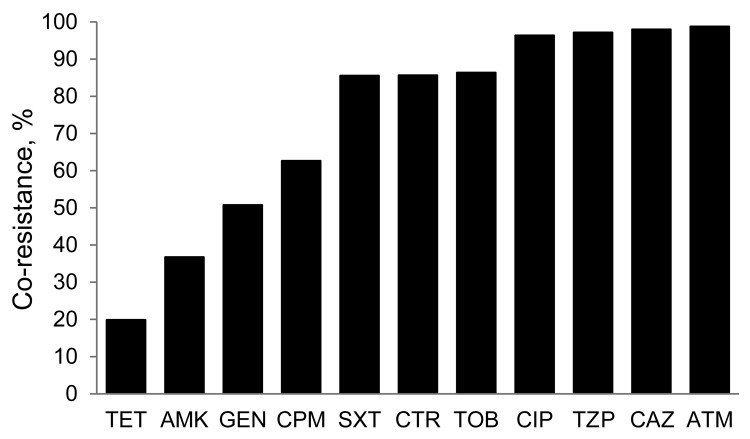
Prevalence of antimicrobial cross-resistance among imipenem-resistant *Klebsiella pneumoniae* isolates, United States, 2010. TET, tetracycline; AMK, amikacin; GEN, gentamicin; CPM, cefepime; SXT, trimethoprim/sulfamethoxazole; CRO, ceftriaxone; TOB, tobramycin; CIP, ciprofloxacin; TZP, piperacillin/tazobactam; CAZ, ceftazidime; ATM, aztreonam.

Imipenem-resistant isolates of *K. pneumoniae* showed the lowest resistance to tetracycline (19.9%) and amikacin (36.8%). High prevalence of cross-resistance was observed for ciprofloxacin (96.4%) (Figure 2).

## Conclusions

In our study, the proportion of *K. pneumoniae* isolates resistant to carbapenems was lower than those previously reported (*7*,*8*). In 2010, we observed a resistance rate of 4.3% for imipenem. The Centers for Disease Control and Prevention (CDC) reported that, among health care–associated infections, 8% of *Klebsiella* spp. isolates were carbapenem resistant in 2007 compared with <1% in 2000 (*9*). Most studies of *K. pneumoniae* antimicrobial drug resistance have focused on patient populations with higher exposures to antimicrobial agents, such as those in critical care and academic hospital settings. In contrast, the lower prevalence of CRKP in our study might have resulted from a wider variety of institution types and inclusion of isolates from hospital patients outside of the critical care setting. Furthermore, within our study, a high percentage of isolates were from urine and showed lower levels of resistance than did isolates from respiratory samples. Interpretive breakpoint criteria for the antimicrobial agents included did not change during the study period.

The low cross-resistance to tetracycline among CRKP and stable resistance rate of *K. pneumoniae* to this agent during the study period are noteworthy. In our analysis of cross-resistance among imipenem-resistant *K. pneumoniae*, tetracycline had the greatest antimicrobial activity against CRKP. Although resistance of *K. pneumoniae* increased for all antimicrobial agents studied, resistance to tetracycline increased only slightly from 1998 to 2010. Later-generation tetracyclines may prove useful in the treatment of CRKP-related infections because of their improved tissue penetration, antimicrobial activity, and decreased propensity to develop antimicrobial drug resistance compared with their older counterparts (*10*). Tigecycline, a glycylcycline antimicrobial agent that is structurally similar to tetracycline, has been used to treat CRKP-related infections and is often active against carbapenemase–producing *K. pneumoniae* (*11*,*12*). Data for tigecycline that used FDA interpretive breakpoints showed *K. pneumoniae* antimicrobial drug resistance was 2.6% in 2010. Tigecycline data were included only for 2010 because the drug was not FDA approved until 2005 and an insufficient number of results were available before 2010.

The widespread transmission of carbapenemase-producing *K. pneumoniae* has become the most common cause of carbapenem resistance among *Enterobacteriaceae* in the United States (*13*) and probably accounts for most of the imipenem resistance shown in this study. The spread of carbapenemase-producing organisms threatens to extend carbapenem resistance to the community (*14*). The increasing antimicrobial drug resistance to *K. pneumoniae* in our study, a concurrent lack of novel antimicrobial agent development (*15*), and limited therapeutic options available for treating CRKP-related infections add further urgency to improve prevention efforts and treatment strategies.

Our study data have strengths and limitations. The strengths are the wide variety of antimicrobial agents included, the number of laboratories reporting data, the nationally representative geographic distribution of these institutions, and the large number of isolates. Geography is a critical consideration with surveillance of this organism because distribution of *K. pneumoniae* antimicrobial drug resistance varies within the United States (*13*). The limitations of these data include a lack of central laboratory testing and the variety of test methods used. Because of a lack of Clinical Laboratory Standards Institute or FDA interpretive breakpoints for *K. pneumoniae* and colistin or fosfomycin, these data were not collected by TSN Database-USA and were not included in this study. Resistance to carbapenems might have been underreported at the beginning of our study period because of a lower frequency of susceptibility testing of these agents and the inability of antimicrobial susceptibility test methods to detect low-level carbapenem resistance.

Our study shows that *K. pneumoniae* antimicrobial drug resistance increased for every antimicrobial class studied except tetracyclines. Cross-resistance among imipenem-resistant *K. pneumoniae* was high for ciprofloxacin but lower for tetracycline and amikacin. This emerging problem presents a major threat to public health and warrants due diligence in future surveillance efforts.

Technical Appendix *Klebsiella pneumoniae* imipenem resistance among ICU and non-ICU isolates, United States, 1998–2010.
