# Supplementary material for: Klebsiella pneumoniae Antimicrobial Drug Resistance, United States, 1998–2010
Source: Emerg Infect Dis. 2013 Jan;19(1):133–6. doi: 10.3201/eid1901.120310 (PMC3557979; doi:10.3201/eid1901.120310)
Supplement: Technical Appendix — Klebsiella pneumoniae imipenem resistance among ICU and non-ICU isolates, United States, 1998–2010. [file 12-0310-Techapp-s1.pdf]

Article DOI: http://dx.doi.org/10.3201/eid1901.120310

Klebsiella pneumoniae Antimicrobial Resistance, United States, 1998–2010

Technical Appendix

Technical Appendix Table. *Klebsiella pneumoniae* imipenem resistance among ICU and non-ICU isolates, United States, 1998–2010*

Setting	1998	1999	2000	2001	2002	2003	2004	2005	2006	2007	2008	2009	2010
ICU	0.0%	0.0%	0.0%	0.0%	0.0%	0.1%	0.3%	0.8%	0.4%	2.1%	4.4%	4.9%	6.3%
Non-ICU	0.0%	0.0%	0.0%	0.0%	0.0%	0.0%	0.3%	0.5%	0.7%	1.3%	3.0%	3.5%	3.8%

*ICU, intensive care unit.
